# Improving Pediatric Follow-Up Documentation Through a Structured Follow-Up Card: A Closed-Loop Quality Improvement Project in Sudan

**DOI:** 10.7759/cureus.108433

**Published:** 2026-05-07

**Authors:** Baraa Mohamed Awadalla Mohamed, Egbal Sahal Abdelmaged Ibrahim, Hibatalla Nasreldeen Khalid Siedahmed, Hala Mubarak Ahmed Dafalla, Albashir Alkabashi Albashir Almsbah, Abrar Abdelghani Mohammed Salih, Hajir Jamal Salman Abdalgani, Sittana Adam Murdas, Salma Gumaa Dayein Hayo, Nada Salaheldin Suliman Ali, Rayan Khalid Ahmed Malik, Sara Osman Salih Elmatari, Abrar Mustafa Albashir Osman, Wissal Fatih, Aisha Hassab Elrasoul Osman Mohammed, Abdalmahmoud Asadig Kanan Ahmed, Amna Mohamed Hassan, Rahmat Abiola Abdulazeez, Wishah Busati Saad Mohamed, Israa Mohammed Alfatih Hussein Albadawy

**Affiliations:** 1 Medical Education, Hamad Medical Corporation, Doha, QAT; 2 Pediatrics Department, Almanagil Teaching Hospital, Al Managil, SDN; 3 Internal Medicine, Dongola Specialized Hospital, Dongola, SDN; 4 Emergency Department, Almanagil Teaching Hospital, Al Managil, SDN; 5 Emergency Department, Burjeel Hospital, Abu Dhabi, ARE; 6 Surgery Department, Almanagil Teaching Hospital, Al Managil, SDN; 7 Internal Medicine and General Surgery Department, Almanagil Teaching Hospital, Al Managil, SDN; 8 Emergency Medicine, Atbara Teaching Hospital, Atbara, SDN

**Keywords:** almanagil teaching hospital, follow-up records, pediatric practice, resource-limited settings, sudan

## Abstract

Background

Accurate and complete clinical documentation is essential for ensuring patient safety, continuity of care, and effective clinical decision-making. In many resource-limited settings, documentation practices remain inconsistent due to the absence of standardized tools, leading to gaps in care delivery.

Objective

This study aimed to evaluate and improve the completeness of pediatric follow-up documentation through the implementation of a structured follow-up card.

Methods

This was a closed-loop quality improvement project conducted at the pediatric outpatient department of Almanagil Teaching Hospital, Sudan. Two audit cycles were performed. The first cycle involved a retrospective review of 50 pediatric follow-up records over a two-week period in August 2025 to assess baseline documentation practices. Following this, a one-month intervention was implemented, introducing a structured pediatric follow-up card alongside brief staff orientation. The second cycle was conducted prospectively over two months (October-November 2025), including 50 follow-up records. Documentation completeness was assessed using predefined criteria, and comparisons between cycles were performed using the chi-square test, with a p-value <0.05 considered statistically significant.

Results

At baseline, documentation across all assessed variables was absent (0%). Following the intervention, significant improvements were observed across nearly all variables. Core documentation elements, including patient identification details, diagnosis, and treatment plan, reached 100% compliance (n = 50). Clinical variables such as chronic disease status and medication-related documentation improved to 74.0% (n = 37) and 68.0% (n = 34), respectively, while medication frequency and nutritional documentation exceeded 98.0% (n = 49). All improvements were statistically significant (p < 0.001), except for medical file number documentation, which remained unchanged.

Conclusion

The introduction of a structured pediatric follow-up card significantly improved documentation completeness. This study highlights the effectiveness of simple, low-cost interventions in addressing system-level documentation gaps and improving the quality of care in resource-limited settings.

## Introduction

High-quality clinical documentation is a fundamental component of safe and effective healthcare delivery, serving as a cornerstone for communication, continuity of care, and clinical decision-making. Inadequate or inconsistent documentation has been widely associated with fragmented care, medical errors, and suboptimal patient outcomes, particularly in resource-limited settings where reliance on paper-based systems remains prevalent [1,2]. In pediatric practice, the importance of accurate documentation is further amplified due to the dependency on caregivers, the need for longitudinal follow-up, and the complexity of developmental and preventive care requirements.

Continuity of care is a critical determinant of healthcare quality, especially in pediatric populations, where ongoing monitoring, vaccination schedules, and growth assessment require consistent and structured follow-up. Evidence suggests that improved continuity is associated with better clinical outcomes, enhanced patient satisfaction, and reduced healthcare utilization, particularly among children with chronic or complex conditions [3,4]. However, maintaining continuity remains challenging in many healthcare systems due to gaps in communication and the absence of standardized documentation tools.

Structured documentation tools, such as standardized forms and clinical templates, have been shown to significantly improve the completeness, clarity, and usability of medical records. By reducing variability in documentation practices, these tools enhance interdisciplinary communication and facilitate coordinated care delivery [2]. Quality improvement (QI) initiatives focusing on structured documentation have demonstrated substantial improvements in documentation rates, with some studies reporting increases from baseline levels as low as 0% to over 80% following targeted interventions [5]. These findings highlight the effectiveness of simple, low-cost interventions in addressing systemic documentation deficiencies.

In pediatric settings, the use of structured follow-up or care continuity tools is particularly important in bridging the gap between healthcare providers and caregivers. Effective documentation not only guides clinical management but also empowers caregivers with essential information regarding diagnosis, treatment, and follow-up plans. Studies have emphasized that well-designed documentation systems improve care coordination and support family-centered care, which is central to pediatric practice [6]. Conversely, the absence of such systems may lead to missed follow-up appointments, medication errors, and poor adherence to treatment plans.

Despite the recognized importance of structured documentation, many healthcare facilities in low- and middle-income countries continue to rely on unstandardized or incomplete recording practices. This results in variability in documentation quality and limits the ability to ensure continuity of care across different encounters and providers [7]. Addressing these gaps through targeted QI interventions is essential to improving patient safety and healthcare outcomes.

This study aimed to evaluate and improve the quality of pediatric follow-up documentation at Almanagil Teaching Hospital through the implementation of a standardized follow-up card. The primary outcome was the proportion of records with complete documentation across predefined variables. Using a closed-loop audit design, the project assessed baseline documentation practices, introduced a structured intervention, and re-evaluated performance to determine the effectiveness of the implemented changes.

## Materials and methods

This study was conducted as a closed-loop QI project using a two-cycle clinical audit design to evaluate and enhance the quality of pediatric follow-up documentation at Almanagil Teaching Hospital, Sudan. The project was structured in accordance with established QI methodology, incorporating baseline assessment, targeted intervention, and re-evaluation to measure the effectiveness and sustainability of change.

The study was carried out in the pediatric outpatient and follow-up clinics over a defined period from August to November 2025. The first audit cycle was conducted over a two-week period in August 2025 and involved a retrospective review of routinely completed pediatric follow-up records to assess baseline documentation practices.

At the time of the baseline audit, the department did not utilize a standardized pediatric follow-up card or structured documentation format. Follow-up visits were conducted in an unstructured manner, with patients typically presenting without a dedicated follow-up record, and clinical information was often communicated verbally by caregivers or documented inconsistently in non-standardized notes. Consequently, when assessed against predefined documentation criteria, all variables were recorded as absent (0%) at baseline, reflecting a system-level absence of structured documentation rather than absence of clinical care delivery. This was followed by a one-month intervention phase during September 2025. The second audit cycle was then conducted prospectively over a two-month period from October to November 2025 using the newly implemented documentation system.

The study population included pediatric patients attending follow-up visits during the audit periods. All eligible follow-up records within the defined timeframes were included using a consecutive sampling approach to reflect routine clinical practice. Records that lacked sufficient information to assess key documentation variables were excluded from the analysis.

A structured data collection tool was developed based on predefined standards for pediatric clinical documentation and continuity of care. The tool captured essential variables, including patient identification details, clinical assessment findings, diagnosis, treatment plan, medication details, follow-up instructions, and healthcare provider identification. Documentation completeness was defined as the presence of each predefined variable within the clinical record. Each variable was assessed as a binary outcome (documented or not documented), enabling objective evaluation of compliance with documentation standards.

Baseline data from the first audit cycle were analyzed to identify deficiencies in documentation practices. Based on these findings, a targeted intervention was designed and implemented. This consisted of introducing a standardized pediatric follow-up card aimed at improving the completeness, clarity, and consistency of documentation. The follow-up card included predefined fields covering patient identification (name, age, and gender), clinical assessment (diagnosis and chronic conditions), medication details (dose, frequency, and duration), nutritional status, follow-up instructions, and healthcare provider identification. The card was designed to be simple, practical, and aligned with existing clinical workflows to facilitate routine use without disrupting service delivery.

Healthcare providers were oriented to the use of the follow-up card through brief on-site educational sessions, which included an explanation of each documentation field, a demonstration of correct completion, and clarification of documentation standards. Visual aids and ongoing informal feedback were also used during the intervention phase to reinforce adherence and promote sustained use of the tool.

Following the intervention, the second audit cycle was conducted using the same data collection tool and assessment criteria to ensure consistency and comparability between cycles. Data were collected prospectively from completed follow-up cards and analyzed to assess changes in documentation performance.

Statistical analysis was performed using appropriate descriptive and inferential methods. Categorical variables were summarized as frequencies and percentages. Comparisons between the first and second audit cycles were conducted using the chi-square (χ²) test to assess differences in documentation rates. A p-value of less than 0.05 was considered statistically significant.

This project was conducted as a QI initiative at the pediatric outpatient department of Almanagil Teaching Hospital, Sudan, and received institutional ethical approval under reference number ATH/IRB/2025/022. The study was designed to enhance routine clinical documentation practices and did not involve any direct patient intervention or alteration of standard clinical care. No patient-identifiable data were collected, and all information was anonymized prior to analysis to ensure confidentiality.

Institutional approval for conducting the audit was obtained from the hospital administration, and the project was carried out in accordance with the ethical principles governing QI activities and the Declaration of Helsinki.

## Results

A total of 100 pediatric follow-up records were analyzed across two audit cycles, with 50 records reviewed in each cycle. At baseline (Cycle 1), documentation across all assessed variables was uniformly absent, with 0% completion recorded for all parameters. This finding reflects the absence of a structured follow-up documentation system during the pre-intervention phase, where no standardized follow-up card was in use and clinical information was not routinely documented in a consistent or retrievable format.

Following the implementation of the structured pediatric follow-up card, substantial improvements were observed across nearly all documentation domains in Cycle 2. Core patient identification variables, including patient name, age, and gender, improved from 0% to 100% (n = 50 for each). Similarly, key clinical and administrative elements, such as diagnosis, doctor’s name, clinic name, and date of visit, also achieved full documentation rates of 100% (n = 50), all demonstrating statistically significant improvements (χ² = 100.0, df = 1, p < 0.001).

Clinical care-related documentation showed marked but variable improvement. Documentation of chronic diseases increased to 74.0% (n = 37), while medication allergies and food allergies were recorded in 68.0% of cases (n = 34 each). Nutritional documentation, including the age of starting meals, also reached 68.0% (n = 34), whereas the number of meals was documented in 98.0% of cases (n = 49). All these improvements were statistically significant (df = 1, p < 0.001).

Medication-related documentation demonstrated substantial gains, with medication prescription and duration documented in 100% of cases (n = 50), frequency of medication in 98.0% (n = 49), and dose of medication in 74.0% (n = 37). In addition, documentation of emergency warning symptoms, treatment plans, and facilitator name reached full compliance (100%, n = 50 for each), all with statistically significant improvements (df = 1, p < 0.001). Signature documentation also improved markedly to 96.0% (n = 48).

By contrast, documentation of the medical file number showed no improvement, remaining at 0% in both audit cycles.

Overall, the implementation of the structured follow-up card resulted in a significant and consistent improvement in documentation completeness across the majority of assessed variables, with most parameters achieving near-complete or complete compliance in the second audit cycle (Table 1).

**Table 1:** Comparison of documentation completeness between audit cycles Comparison of documentation completeness across assessed variables between the first and second audit cycles. Values are presented as frequency (n) and percentage (%). Statistical analysis was performed using the chi-square test.

Variable	Cycle 1 n (%)	Cycle 2 n (%)	χ²	df	p-value
Patient name	0 (0%)	50 (100%)	100.0	1	<0.001
Patient age	0 (0%)	50 (100%)	100.0	1	<0.001
Patient gender	0 (0%)	50 (100%)	100.0	1	<0.001
Medical file number	0 (0%)	0 (0%)	—	—	—
Diagnosis	0 (0%)	50 (100%)	100.0	1	<0.001
Doctor’s name	0 (0%)	50 (100%)	100.0	1	<0.001
Clinic name	0 (0%)	50 (100%)	100.0	1	<0.001
Date of visit	0 (0%)	50 (100%)	100.0	1	<0.001
Chronic diseases	0 (0%)	37 (74.0%)	58.7	1	<0.001
Medication allergies	0 (0%)	34 (68.0%)	52.6	1	<0.001
Vaccination status	0 (0%)	50 (100%)	100.0	1	<0.001
Food allergies	0 (0%)	34 (68.0%)	52.6	1	<0.001
Age of starting meals	0 (0%)	34 (68.0%)	52.6	1	<0.001
Number of meals	0 (0%)	49 (98.0%)	96.0	1	<0.001
Medication prescribed	0 (0%)	50 (100%)	100.0	1	<0.001
Dose of medication	0 (0%)	37 (74.0%)	58.7	1	<0.001
Frequency of medication	0 (0%)	49 (98.0%)	96.0	1	<0.001
Duration of medication	0 (0%)	50 (100%)	100.0	1	<0.001
Emergency warning symptoms	0 (0%)	50 (100%)	100.0	1	<0.001
Treatment plan	0 (0%)	50 (100%)	100.0	1	<0.001
Facilitator name	0 (0%)	50 (100%)	100.0	1	<0.001
Signature	0 (0%)	48 (96.0%)	92.2	1	<0.001

## Discussion

The findings of this study demonstrate a substantial and statistically significant improvement in the completeness of pediatric follow-up documentation following the implementation of a structured follow-up card. The transition from complete absence of documentation at baseline to near-complete or complete compliance across most variables highlights the critical role of structured documentation tools in addressing systemic deficiencies in clinical record-keeping. Importantly, the complete absence of documentation observed at baseline reflects the lack of a structured follow-up documentation system in the study setting, where no standardized follow-up card was in use and clinical information was not routinely recorded in a consistent or retrievable format, rather than an absence of clinical care delivery. These findings reinforce the concept that documentation gaps in clinical practice are often not due to lack of knowledge alone, but rather reflect system-level issues such as the absence of standardized tools, unclear expectations, and workflow inefficiencies.

The observed improvements are consistent with a growing body of international evidence demonstrating that structured documentation templates significantly enhance the quality, completeness, and usability of medical records. Standardized formats, such as SOAP-based or checklist-driven documentation, have been shown to improve interprofessional communication, reduce medical errors, and enhance patient safety by ensuring that essential clinical information is consistently recorded [8,9]. In pediatric settings, where continuity of care and caregiver understanding are particularly important, structured documentation becomes even more critical, as it supports longitudinal monitoring and facilitates clear communication between healthcare providers and families.

Importantly, the magnitude of improvement observed in this study is comparable to, and in some domains exceeds, findings reported in similar QI initiatives conducted in resource-limited settings. A clinical audit in pediatric surgery at the National Ribat University Hospital demonstrated that the introduction of structured documentation formats, combined with training and audit feedback, led to compliance rates exceeding 90% in key documentation domains [8]. Similarly, a QI project at Dongola Specialized Hospital reported an increase in documentation completeness from 31.1% to 84.9% following the implementation of a standardized template and targeted educational interventions [10]. These parallels highlight that the effectiveness of such interventions is reproducible across different clinical contexts within Sudan, reinforcing the external validity of the present findings.

The findings of this study also reflect broader systemic challenges in documentation practices within Sudan and comparable low- and middle-income countries. Previous studies conducted in Khartoum hospitals have demonstrated that although healthcare providers often possess adequate theoretical knowledge, the quality of documentation remains suboptimal due to the lack of formal guidelines, structured formats, and continuous training [11]. Similarly, national-level analyses emphasize that deficiencies in documentation are closely linked to weaknesses in health information systems, the absence of standardized protocols, and limited quality assurance mechanisms [12]. The complete absence of documentation observed in the first audit cycle of the present study aligns with these systemic gaps, further supporting the argument that documentation quality is primarily driven by organizational factors rather than individual clinician performance alone.

A key strength of the intervention in this study lies in its simplicity and contextual adaptability. The structured follow-up card was designed to integrate seamlessly into the routine clinical workflow, minimizing disruption while maximizing usability. This aligns with evidence suggesting that low-cost, context-appropriate interventions-such as paper-based templates, visual cues, and brief educational sessions-can achieve substantial improvements in documentation quality even in resource-constrained environments [13]. Furthermore, the use of a closed-loop audit design enabled not only the identification of baseline deficiencies but also the evaluation of intervention effectiveness, which is considered a best practice in QI methodology.

Despite the overall success of the intervention, some variability in documentation completeness persisted across certain variables, particularly those requiring more detailed clinical input, such as chronic disease status, allergy documentation, and medication dosing. These findings are consistent with previous studies, which have shown that more complex or cognitively demanding documentation elements are less consistently completed compared to simpler demographic or administrative fields [13]. Potential contributing factors include time constraints, workflow pressures, and the absence of mandatory prompts or reinforcement mechanisms for these specific variables. Addressing these gaps may require further refinement of the documentation tool, including the incorporation of visual cues, mandatory fields, or additional training focused on high-risk documentation areas.

The lack of improvement in the documentation of medical file numbers represents an important and noteworthy finding. This may reflect system-level barriers beyond the scope of the intervention, such as issues related to patient registration processes or the availability of standardized identifiers. Similar challenges have been reported in other studies, where certain documentation elements remain resistant to improvement due to structural or administrative limitations rather than clinician behavior [12]. This highlights the importance of adopting a systems-based approach to QI, recognizing that not all gaps can be addressed through clinical interventions alone.

From a broader perspective, this study underscores the critical role of structured documentation in enhancing patient safety, continuity of care, and overall healthcare quality. Accurate and complete clinical records serve not only as a communication tool but also as a foundation for clinical decision-making, audit, research, and medico-legal accountability. Inadequate documentation has been shown to negatively impact patient management, increase the risk of medical errors, and compromise continuity of care, ultimately affecting patient outcomes [14].

Furthermore, evidence from systematic reviews indicates that structured and standardized documentation systems significantly improve documentation quality, clarity, and usability, thereby supporting safer and more effective clinical care [15]. The significant improvements observed in this study support the scalability of similar interventions across other departments and healthcare facilities, particularly in resource-limited settings where electronic health systems may not be readily available.

However, the sustainability of these improvements remains a key consideration. Evidence suggests that without ongoing reinforcement, audit cycles, and institutional support, gains in documentation quality may diminish over time [8]. Therefore, maintaining the improvements observed in this study will likely require continued education, regular monitoring, and integration of the structured documentation tool into routine clinical governance frameworks.

Despite the significant improvements observed, this study has several limitations that should be acknowledged. First, the study was conducted in a single center, which may limit the generalizability of the findings to other healthcare settings with different patient populations, resources, or organizational structures. Second, the relatively modest sample size, although adequate for a QI audit, may limit the statistical power and precision of the estimates, particularly for variables with partial compliance. Third, the use of a before-and-after audit design without a control group introduces the possibility that external factors, such as increased staff awareness or temporal changes in practice, may have contributed to the observed improvements. Additionally, data collection relied on documented records, which may be subject to documentation bias, as improved recording does not necessarily reflect a corresponding improvement in actual clinical practice. Observer bias may also have been present, as data collectors were not blinded to the audit cycle due to the visible differences in documentation format.

Furthermore, the short interval between the intervention and the re-audit limits the ability to assess the long-term sustainability of the observed improvements. Evidence from QI literature suggests that without continuous reinforcement, monitoring, and institutional support, improvements in documentation practices may decline over time. Finally, certain variables, such as medical file number documentation, were not responsive to the intervention, highlighting the presence of system-level barriers that were beyond the scope of this study and require broader organizational or administrative solutions.

## Conclusions

The implementation of a structured pediatric follow-up card was associated with a significant improvement in documentation completeness across most assessed variables, underscoring the importance of standardized documentation tools in addressing system-level gaps and enhancing the quality and consistency of clinical records. While high levels of compliance were achieved in most domains, the persistence of variability in certain clinical variables and the lack of improvement in documenting medical file numbers highlight the need for ongoing monitoring and targeted system-level interventions. Overall, these findings demonstrate that simple, low-cost, and context-appropriate strategies can effectively improve documentation practices, thereby supporting safer clinical processes and improved continuity of care, particularly in resource-limited settings.
